# Assessment of the Impact of COVID-19 on Drug Store Management in a Tertiary Care Teaching Hospital of Central India

**DOI:** 10.7759/cureus.19723

**Published:** 2021-11-18

**Authors:** Srikanta Padhan, Pugazhenthan T, Ramesh Chandrakar, Abhiruchi Galhotra, Nitinkumar B Borkar

**Affiliations:** 1 Epidemiology and Public Health, All India Institute of Medical Sciences, Raipur, IND; 2 Pharmacology and Therapeutics, All India Institute of Medical Sciences, Raipur, IND; 3 Tranfusion Medicine and Blood Banking, All India Institute of Medical Sciences, Raipur, IND; 4 Paediatrics Surgery, All India Institute of Medical Sciences, Raipur, IND

**Keywords:** utilization, shortage, impact, drug store, pandemic, antibiotics

## Abstract

Introduction: One-third of the annual hospital budget is spent on the purchase of medicines, materials, and supplies. Drug store management is a complex but critical process within the healthcare delivery system. Health supply chains, the import of active pharmaceutical ingredients, transportation, procurement, finished products have been disrupted by COVID-19.

Materials & methods: A retrospective, observational study was carried out at the Department of Hospital Administration, All India Institute of Medical Sciences (AIIMS), Raipur. Quantitative data about the pattern of consumption of 20 most commonly used drugs (10 antibiotics, three analgesics, three antipyretics, two anticoagulants, and two steroids), and 20 most frequently used consumables were sourced from existing records of the Central Pharmacy for 24 months between 1st January 2019 to 31st December 2020.

Results: A significant rise in the consumption pattern was seen in 25 drugs and consumables out of 40 total selected drugs and consumables. The maximum increase was observed in antibiotics followed by antipyretics, and the least increase was observed in analgesics followed by anticoagulants. Tablet Azithromycin 500 mg was the most frequently used antibiotic during the COVID-19 Period as compared to the Pre-COVID-19 period followed by injection Piperacillin + Tazobactam. The only antibiotic having a decline in consumption and also with the lowest consumption was tablet Metronidazole 400 mg. The highest increase in consumables occurs by 10088% in N95 Masks, followed by 573% in shoe covers, and 153% in face masks (three-layers), respectively.

Conclusion: This study will enhance education to the pharmaceutical industries, policymakers to the Government, and other hospitals on how to better manage drug stores in future pandemic-like situations. Proper drug store management played a crucial role in medication usage that improved patient outcomes and prevented the misuse of medications. The pattern of changes in the consumption of drugs and consumables in the present study can be utilized by other hospitals in the third wave of the pandemic.

## Introduction

The drug store is one of the hospital's most widely used treatment plants and one of the few areas that regularly spend a considerable amount of money on purchases. About one-third of the annual hospital budget is spent on the purchase of medicines, materials, and supplies [1]. The new coronavirus strain is a member of a large family of viruses that cause a wide range of respiratory illnesses ranging from the common cold to more severe disorders such as pneumonia [2]. The COVID-19 pandemic has quickly altered our daily lives, enterprises, and disrupted world trade and movements. As COVID-19 continues to spread, supply chains and logistics vulnerabilities have been exposed [3]. During the initial phase, it rendered many drugs and consumables unavailable. Health supply chains, active pharmaceutical ingredients, transportation, procurement, finished products, and many more have been disrupted [4].

Management of stock (drugs and consumables) ensures availability and minimizes investment [5]. Drug store management is a complex but critical process within the healthcare delivery system [6]. Hospitals risk being unable to give patients the most suitable medication at the right time without efficient drugstore management practices. Additionally, pharmacy dispensing patterns and drug selection choices may have a direct effect on the quality of patient care [7,8]. In addition to the safety of patients and financial considerations, the management of the drug store also raises the importance of keeping effective monitoring of drug stocks in today's growing healthcare environment [9]. During Lockdown, all hospital outpatient departments (OPDs) were closed, some diagnostic procedures and routine surgeries were postponed, and most of the work in the hospital was mainly related to the management of COVID-19 patients. In such a situation, stock-outs of essential items could severely impede patient care and hospital operations, resulting in an unacceptable negative impact on patient outcomes [10].

Drug utilization is referred to as “the marketing, distribution, prescription, and use of drugs in a society with special emphasis on the resulting medical and social consequences” [11]. It is an important tool for the study and impact on the health system of clinical consumption of drugs in populations [12,13]. The rationale for conducting this study was to avoid stock-outs and to stockpile the drugs and consumables in the hospital by estimating the surge/decline in drugs and consumables utilization after COVID-19, and to develop a proper management plan for the drug store to deal with a similar pandemic situation in future. Also, there was a need to measure the underutilized stock quantity left due to COVID-19.

## Materials and methods

A retrospective, observational study was carried out at the Department of Hospital Administration, AIIMS Raipur. Ethical clearance for this study was obtained from the Institute Ethics Committee of All India Institute of Medical Sciences, Raipur (Ref No: 1488/IEC-AIIMSRPR/2021). A total of 20 most commonly used drugs (10 antibiotics, three analgesics, three antipyretics, two anticoagulants, and two steroids) and 20 most frequently used consumables were selected based on the last two-year consumption rate from central pharmacy data. Quantitative data on the pattern of demand, supply and consumption of drugs, and hospital consumables were sourced from the existing records of the central pharmacy for 24 months from 1st January 2019 to 31st December 2020.

To assess the potential impact of the COVID-19 pandemic on drug store management, the study period was separated into two periods: a pre-pandemic or pre-COVID-19 period (1st January 2019 to 31st December 2019), and the COVID-19 pandemic period (1st January 2020 to 31st December 2020). However, we acknowledge that the first confirmed case of COVID-19 was admitted on the 18th March 2020 in our hospital and the first COVID-19 case in India was reported on 27th January 2020; the data for the whole month of January was included. After calculating the monthly consumption for each month, the mean average monthly consumption in the pre-COVID-19 period (2019) and the mean average monthly consumption during the COVID-19 period (2020) were calculated separately.

The data of stock and monthly consumption of selected drugs and consumables were collected from the central pharmacy in a predesigned proforma. The data regarding total functional beds, average occupied beds, and the average bed occupancy rate was collected from the Medical Record Department (see Appendix A). The drug consumption and utilization pattern of the most commonly used drugs in the hospital were studied. Most commonly used drugs were classified according to the anatomical therapeutic chemical (ATC) classification system, and drug utilization was measured in DDD/100 bed-days.

The data was transcribed in an MS Excel spreadsheet (Microsoft Corporation, Redmond, WA) in the form of frequency, percentages, and graphs. Data analysis was carried out using SPSS V26 statistical software (IBM Corp., Armonk, NY). A Chi-square test was used for comparison. A p-value of <0.05 was taken as statistically significant. Drug utilization pattern was analyzed by comparing mean utilization rate of pre-COVID-19 period and during COVID-19 period by paired t-test, and p-value of <0.05 was considered to be a significant mean difference.

## Results

We observed that out of 20 selected drugs, there was a surge in drug consumption of 15 drugs and a decline in drug consumption in only five drugs in 2020 when compared to 2019.

Also, out of the 20 selected consumables, there was a surge in consumption of 10 consumables and a decline in the consumption of nine other consumables, and no change in the consumption of only one consumable in 2020 as compared to 2019.

**Figure 1:**
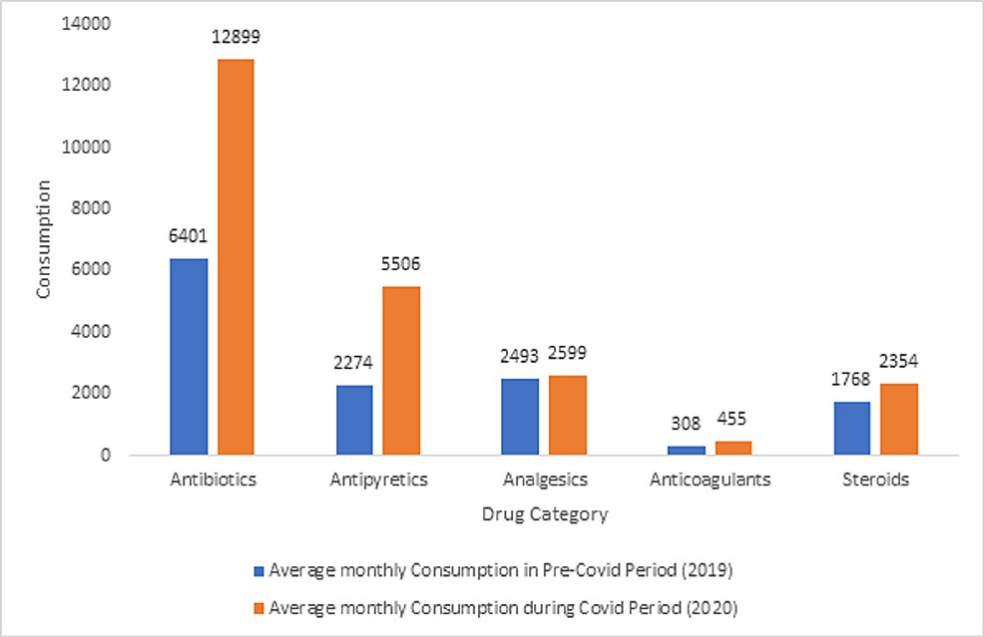
Category-wise drug consumption pattern

Figure 1 shows that there was an overall increase in drug consumption during COVID-19 in five categories of drugs. The maximum increase occurs in antibiotics followed by antipyretics. The least increase occurs in analgesics followed by anticoagulants.

Table 1 shows that before COVID-19, the five most commonly consumed antibiotics were the injection Piptaz, tablet metronidazole 400 mg, injection metronidazole 100 ml, tablet azithromycin 500 mg, and tablet ceftriaxone 500 mg, respectively. During the pandemic, the five most commonly consumed antibiotics were tablet azithromycin 500 mg, injection piperacillin + tazobactam, injection ceftriaxone 1 gm, injection metronidazole 100 ml, and tablet ceftriaxone 500 mg. The highest surge in consumption was for the antibiotic injection ceftriaxone 1 gm (273%) followed by azithromycin 250 mg (204%) with p <0.05. The only antibiotic having a significant decline in consumption was tablet metronidazole 400 mg (-36 %).

**Table 1:** Change in overall drug consumption pattern

Drug/Consumable Name	Total Consumption in Pre-COVID-19 Period (2019)	Total Consumption During COVID-19 Period (2020)	Increase/Decrease (%)	Chi-square Value	P-value
Injection piperacillin 4 gm + tazobactam 500 mg	16393	30292	85	3815.61	P <0.00001
Tablet metronidazole 400 mg	12635	8035	-36	1387.12	P <0.00001
Injection metronidazole 100 ml	11279	19305	71	15390.79	P <0.00001
Tablet azithromycin 500 mg	11100	30300	173	203.05	P <0.00001
Injection ceftriaxone 500 mg	6400	16300	155	1067.90	P <0.00001
Injection meropenem 1 gm	5588	7626	36	2422.33	P <0.00001
Injection amoxicillin + clavulanic acid 1.2 gm	5420	9862	82	998.75	P <0.00001
Injection ceftriaxone 1 gm	5310	19810	273	4965.88	P <0.00001
Tablet azithromycin 250 mg	3260	9900	204	238.62	P <0.00001
Tablet ciprofloxacin 500 mg	3180	6310	98	624.5	P <0.00001
Tablet paracetamol 500 mg	17090	53210	211	1730.61	P <0.00001
Injection paracetamol 2 ml	7135	6865	-4	1482.62	P <0.00001
IV paracetamol 100 ml	3055	6000	96	768.14	P <0.00001
Tablet diclofenac 50 mg	16280	17700	9	2312.68	P <0.00001
Injection diclofenac sodium 1 ml	10000	11160	11.6	0.05	P = 0.4945*
Lignocaine 2% 30 ml	3633	2326	-36	466.98	P <0.00001
Dexamethasone 2 ml	15009	23452	56	283.65	P <0.00001
Hydrocortisone 100 mg	6200	4800	-23	663.89	P <0.00001
Heparin 5000 IU	2082	4500	116	1108.77	P <0.00001
Heparin 25000 IU	1363	965	-29	228.13	P < 0.00001

Table 2 shows that the highest 10821% increase in consumption occurs in N95 face masks followed by shoe covers (624), and triple-layered face masks (172 %) during COVID-19 when compared to the pre-pandemic period with a p-value less than 0.05. The maximum 48% decline in consumption occur in Foley’s catheter 16 followed by syringe 10 ml (34%) and blood glucose strips (25%) during COVID-19 in comparison to the pre-pandemic period (p <0.05).

**Table 2:** Change in overall consumables consumption pattern PPE: Personal protective equipment

Drug/Consumables Name	Total Consumption in Pre-COVID-19 Period	Total Consumption During COVID-19 Period	Increase/Decrease (%)	Chi-square Value	P-value
Face masks (three layers)	544500	1482450	172	3171.18	P <0.00001
Syringes 5 ml	348100	276100	-21	52122.90	P <0.00001
Syringes 10 ml	319150	210850	-34	95323.85	P <0.00001
Syringes 2 ml	316695	288700	-9	28646.55	P <0.00001
Surgical Gloves 7	162350	162545	0	287.42	P <0.00001
Surgical Gloves 7.5	116625	118700	2	199.11	P <0.00001
Cap Male (Surgeon)	136900	203600	49	70627.29	P <0.00001
Cap Female (Buffet)	116500	201750	73	4766.24	P <0.00001
IV Set Micro	16205	22988	42	1282.32	P <0.00001
IV Cannula 22	37790	30280	-20	4497.05	P <0.00001
IV Cannula 20	37522	52448	40	15380.83	P <0.00001
IV Cannula 18	21722	21566	-1	10015.86	P <0.00001
Shoe Cover	22850	165400	624	751.59	P <0.00001
Examination Gloves	14601	21813	49	6693.72	P <0.00001
Urobags	10187	9500	-7	2565.95	P <0.00001
Foley's Catheter 16	5750	2993	-48	806.462	P <0.00001
Foley's Catheter 14	1968	1616	-18	2.90	P =0.88502^*^
Blood Glucose Strips	4316	3224	-25	330.50	P <0.00001
Face Masks N95	1396	152462	10821	1134150.04	P <0.00001
PPE Kit (Disposable)	0	92057	NA	NA	NA

Table 3 shows that dexamethasone 2 ml was the most commonly used drug during the COVID-19 period having a drug consumption of 77.1 defined daily dose (DDD)/100 bed days followed by azithromycin 500 mg with a drug consumption of 56 DDD/100 bed days. There was a significant increase in the amount of utilization of drugs in the COVID-19 period as compared to the pre-COVID-19 period with the highest 189% increase in paracetamol 500 mg utilization followed by a 153 % increase in the consumption of azithromycin 500 mg with p-value less than 0.05. The drugs that had a significant decline in utilization in the COVID-19 period as compared to pre-pandemic period were metronidazole 400 mg with a 40% decline in utilization (p <0.05).

**Table 3:** Analysis of defined daily dose (DDD) per 100 bed days of the drug. Comparison is based on DDD WHO/anatomical therapeutic chemical (ATC) code.

Drug Name	ATC Code	DDD by WHO	DDD/100 Bed Days (2019)	DDD/100 Bed Days (2020)	Increase/Decrease (%)	t Value	p-Value
Piperacillin 4 gm + Tazobactam 500 mg (Oral)	JO1CR05	14 gm	3.4	6.1	79	2.494	0.0298
Metronidazole 400mg (Oral)	JO1XD01	1.5 gm	33.5	19.8	-40	-2.497	0.0296
Meropenem 1 gm (Oral)	JO1DH02	3 gm	1.2	1.5	25	0.185	0.8583^*^
Ceftriaxone 500 mg (Parentral)	JO1DD04	2 gm	3.1	7.5	141	2.412	0.0343
Azithromycin 500 mg (Oral)	JO1FA10	0.5 gm	22.1	56	153	4.408	0.0010
Ciprofloxacin 500 mg (Oral)	JO1MA02	1 gm	10.5	19.4	85	2.139	0.0581^*^
Paracetamol 500 mg (Oral)	NO2BE01	3 gm	18.9	54.6	189	2.855	0.0156
Diclofenac 50 mg (Oral)	MO1AB05	0.1 gm	54	54.5	1	1.388	0.1925^*^
Dexamethasone 2 ml (Parentral)	H02AB02	1.5 mg	53.1	77.1	45	2.620	0.0238
Hydrocortisone 100 mg (Parentral)	HO2AB09	30 mg	13.7	9.8	-28	-0.618	0.5535^*^

Table 4 shows that the average consumption of surgical masks and N95 masks per health care worker per day were 3.70 and 0.37, respectively. This data is based on the peak of the COVID-19 wave in our area during September 2020.

**Table 4:** Consumption comparison of surgical masks and N95 masks at the peak of the COVID-19 wave (September 2020) HCW: Health care worker

Consumables	Total Consumption in September 2020	Average Consumption per Day	Total Health Care Workers	Average Consumption per Day per HCW
Surgical Masks	246750	8225	2220	3.70
N95 Masks	24721	824	2220	0.37

## Discussion

The overall monthly antibiotic usage in 2020 increased significantly (102%) when compared to 2019 (p < 0.0001) especially as the COVID-19 pandemic progressed drastically through March and April 2020. A before and after cross-sectional study conducted by Abelenda-Alonso et al. comparing the data in 2019 and 2020 for the periods from January 1 to April 30 also showed similar results [14]. Another study by Gonzalez-Zorn showed that antibiotic consumption increased significantly by 115% when compared to the 2019 peak. The current study found that the use of azithromycin in 2020 increased by 204%. However, the study conducted by Adriana Ammassari et al. [15] showed a 230% increase in azithromycin use in 2020 compared to 2019. Also, according to another study conducted in Spain [16], there was an increase of 400% in the consumption of azithromycin during the pandemic when compared to the pre-pandemic period. This may be because azithromycin was included in the treatment protocols for the treatment of COVID-19.

The current study shows that some common antibiotics, such as azithromycin and ciprofloxacin, were consumed at their peak during the pandemic's early stages (April to July 2020). The rest of the antibiotics, particularly those with a broader spectrum, such as piperacillin+tazobactam and ceftriaxone, peaked in subsequent months (August to November 2020). This increase could be attributed to an increase in device-related infections (primarily catheter-related bloodstream infections) and superinfections. According to a study conducted by Santiago Grau et al. [17], while the consumption of ceftriaxone and azithromycin increased during March 2020, consumption of daptomycin, carbapenems, linezolid, and ceftaroline increased from April to May 2020.

Except for metronidazole, none of the antibiotics studied in the current study showed a decrease in use when pre-pandemic and pandemic periods were compared. The monthly consumption of antibiotic metronidazole decreased by 36% from April 2020 to August 2020 as COVID-19 restrictions were imposed. The antibiotic metronidazole is mainly used in postoperative patients [18]. The adaptation of Covid appropriate behavior like hand hygiene amongst the population decreased commonly occurring household infections like diarrhea. Also, the closure of operation theatres (OTs) during the lockdown led to a decrease in the consumption of metronidazole. As previously suspended health care services were restored following COVID-19 restrictions, their use gradually returned to baseline.

The monthly consumption of the antipyretic drug paracetamol has increased significantly (211%) during the pandemic period compared to the pre-pandemic period at the hospital. Patients with COVID-19 need special drugs to control lower pain, fever, and inflammation in the early stage. The increase in paracetamol use was driven by guidelines recommended for mild to moderate pain and fever reduction under COVID-19 by public health authorities including the World Health Organization (WHO) [19]. Injection paracetamol is given for fever, but during COVID-19 the clinical staff preferred intravenous over intramuscular to avoid touching the patients as much as possible thus, decreasing its consumption.

As the routine OPDs and inpatient departments (IPDs) were closed from March 2020 to August 2020, there was an overall reduction of 36% in the use of the local anesthetic agent lignocaine at the hospital. The use of anti-inflammatory corticosteroids such as dexamethasone increased by 56% during the study period due to its use in hospitalized patients who require supplemental oxygen. In patients who required mechanical ventilation, the most significant benefit of steroid use was observed. The use of dexamethasone in this context has thus been enormously increased. Also, a decrease of 23% in the consumption of steroid-like hydrocortisone was noticed.

There has been an exponential increase of 172 % in triple-layered surgical masks during the pandemic. Earlier, in the pre-pandemic time, masks were only used in OTs, ICUs, and a few OPDs like in pulmonary medicine, and ENT & medicine, where patients usually present with respiratory complaints. But with the emergence of the COVID-19 pandemic, it was mandatory to use surgical masks for every healthcare worker working in every corner of the hospital; also, the surgical mask was compulsory for asymptomatic COVID-19 patients without oxygen support admitted in the hospital to prevent the infection from spreading to the healthcare workers.

Therefore, a remarkable rise of 10821% in demand and consumption for N95 masks was seen during the pandemic period compared to the pre-pandemic period. To meet the significant volume demand of N95 by the health care sector, the Government of India started mass production and certification by the Bureau of Indian Standard (BIS) under the category of Make in India.

The use of personal protective equipment (PPE) kit is another critical component of the infection control strategy. When there is a risk of infection or exposure to infectious materials, PPE is used [20]. A PPE includes face protection, gloves, gown, headcover, goggles and mask or face shield, and rubber boots if the infection is severe, such as in blood or airborne diseases [21]. It is used to treat diseases such as Ebola and HIV and is now widely used in the treatment of COVID-19. Its primary function is to protect the skin and mucous membranes [22]. While donning and doffing the PPE, the standard operating procedure (SOP) is followed. The current study's findings showed a total of 92057 consumption of coveralls till 31 December 2020 during the COVID-19 pandemic period as it was not used earlier in our institute. 

The consumption of gloves (both surgical and examination) increased during the pandemic as every healthcare provider was using gloves during patient handling, including touching the patient. Also, during Covid care, double gloves are necessary and that too increased the use of surgical and examination gloves. The decline in consumption of consumables was due to the reduction in the number of routine diagnostic and surgical procedures during the pandemic period, especially during the lockdown period.

During the peak of the Covid wave, the adherence to Covid guidelines was almost perfect. The lesser consumption of N95 masks compared to surgical masks was due to restricting its usage to only healthcare providers in highly infectious vulnerable areas as per Indian Council of Medical Research (ICMR) guidelines. The information regarding the consumption of masks per healthcare worker will help in proper demand forecasting for future waves.

Strengths and limitations

The extensive data covering the consumption of a few selected drugs and consumables used in the hospital over a two-year period is the study's strength. This lengthy follow-up period provides valuable insight into the rate of utilization and drug consumption in a tertiary care hospital in a developing country dealing with the challenges of an economic transition in the healthcare system. Another strength of the study is the completeness of the data and the ability to compare data from the same practices across two time periods.

The study has several limitations; It was a single-centered study, i.e., the consumption of drugs and consumables may not be representative of other hospitals in the country. The monthly consumption data was collected from the central pharmacy stock register and there may be a chance of human errors. It is very important to bear in mind during this period that pharmacies work under extremely controlled conditions (e.g., reduced teams) and as the shortages of medicines depend on the direct reports of the pharmacists, the central pharmacy registry may suffer from underreporting. The emergency procurement of drugs and consumables from AMRIT (Affordable Medicines and Reliable Implants for Treatment) is not a continuous process, and there is a lack of information technology during the procurement.

Recommendations

In the fight against the COVID-19 pandemic, the Government should strengthen the drug support services. In hospitals, the unique needs of pharmacy services should be identified and addressed. The hospital administration should ensure that various drugs are available 24 hours a day, seven days a week as they are necessary for patients' care. The cost of drug management and delivery is frequently borne by pharmacy departments. The pharmacy management team should focus on creating effective drug and human resource leverage strategies. The adaptation of a web-based system to monitor the entire logistic and supply chain that includes procurement processes starting from tender processing, then placing purchase orders and stock monitoring, and drug distribution, is essential.

Resource Allocation During a Pandemic

The allocation of resources can be challenging, especially if there is a supply shortage. To address the drug shortage, patient priority criteria should be developed with a multidisciplinary team of medical, nursing, and pharmaceutical staff. Contact with other sites or health systems must be maintained because large health systems are often able to survive drug shortages through a shift in drug stocks between sites.

Medication Storage and Preparation Areas

To guarantee pharmaceutical integrity and personnel safety throughout the hospital, there should be enough facilities for receiving, storing, and preparing pharmaceuticals under sufficient sanitation, temperature, light, moisture, ventilation, segregation, and security conditions. Additional non-functional units of the hospitals should be preserved for the anticipated extra quantity of drugs and consumables during the pandemic.

Maintain an Adequate Supply of COVID-19 Prevention Medications and Products

During the pandemic, most hospitals rely on central pharmacies to adequately supply their medicinal products and COVID-19 preventive products (e.g., masks, alcohol-based hand rubs). Central pharmacies must retain appropriate stocks for supplying demand by pharmaceutical products.

Ensure Safe and Efficient Operation

Central pharmacies shall adopt safe and efficient operations during the pandemic, including appropriate environmental control, personnel protection, and the establishment of an emergency plan. In the face of the COVID-19 pandemic, the pharmacy should also establish new workflows and develop emergency plans or protocols for the management of COVID-19 and possible drug deficiencies.

Use of Automated Systems

All automated pharmacy systems must be evaluated, selected, used, calibrated, monitored, and maintained in accordance with policies and procedures. Drugs and consumables ordering and preparation, drug distribution, and clinical monitoring can all be made safer, more efficient, and more accurate with the help of automated mechanical systems and software.

Use of More Advanced Information Technology

A full-feature pharmacy computer system is required. Other hospital information systems and software, such as computerized provider order entry, medication delivery, electronic health records, and patient billing systems, should be fully networked as well.

Staff Training

Hospitals shall carry out training for all their staff in order to provide pharmacy personnel with sufficient information on the prevention, monitoring, and control of the environment of COVID-19. Guidance must also be included regarding new workflows and emergency plans to deal with pandemics. Adequate pharmacist training is crucial to the successful performance of drug stores.

## Conclusions

The current study is the first that attempts to assess the impact of COVID-19 on drug store management in a tertiary care teaching hospital. A significant rise in the consumption pattern was seen in 25 drugs and consumables out of 40 total selected drugs and consumables. Tablet azithromycin 500 mg was the antibiotic having a maximum rise in consumption during the COVID-19 period followed by injection of piperacillin 4 gm + tazobactam 500 mg. The only antibiotic having a decline in consumption and also with the lowest consumption was tablet metronidazole 400 mg. The highest increase in consumption occurs by 10821 % in N95 Masks, followed by 573 % in shoe covers, and 153 % in surgical masks (three-layered), respectively.

The pattern of changes in drugs and consumables consumption in the present study can be utilized by other hospitals in the third wave of the pandemic. The underutilized drugs and consumables can be returned to the companies or exchanged to prevent their wastage. This study will enhance education to the pharmaceutical industries, policymakers in the Government, and other hospitals, to better manage drug stores in future pandemic-like situations. The pharmaceutical companies should increase their production of overutilized drugs and consumables depending upon the consumption, while the Government should supply the raw materials for the production. Proper drug store management was critical in improving patient outcomes and preventing medication misuse. The long-term consequences of this pandemic are unknown, but they should be closely monitored. More research is needed to determine the COVID-19 pandemic's long-term impact.

## Appendices

Appendix A

The data regarding bed occupancy was collected from the Medical Record Department of AIIMS Raipur.

**Table 5:** Bed occupancy rate from 1st January 2019 to 31st December 2020

Month & Year	Total no. of Functional Beds	Average no. of Occupied beds	Average Bed Occupancy Rate
January 2019	600	306	51.0 %
February 2019	600	321	53.5 %
March 2019	600	337	56.1 %
April 2019	600	335	55.8 %
May 2019	600	348	58.0 %
June 2019	668	410	61.4 %
July 2019	668	450	67.4 %
August 2019	668	453	67.8 %
September 2019	700	482	68.9 %
October 2019	700	465	66.4 %
November 2019	700	522	74.6 %
December 2019	700	531	75.8 %
January 2020	700	534	76.3 %
February 2020	800	536	67.0 %
March 2020	800	403	50.4 %
April 2020	800	211	26.4 %
May 2020	800	317	39.6 %
June 2020	800	365	45.6 %
July 2020	800	409	51.1 %
August 2020	800	510	63.8 %
September 2020	800	562	70.3 %
October 2020	800	450	56.2 %
November 2020	900	498	55.4 %
December 2020	900	531	59.0 %

## References

[1] Devnani M, Gupta A, Nigah R (2010). ABC and VED Analysis of the pharmacy store of a tertiary care teaching, research and referral healthcare institute of India. J Young Pharm.

[2] Abdelrahman Z, Li M, Wang X (2020). Comparative review of SARS-CoV-2, SARS-CoV, MERS-CoV, and Influenza A respiratory viruses. Front Immunol.

[3] Impact of the Pandemic Trade and Development (2021). Impact of the pandemic on trade and development: transitioning to a new normal. Unctad.org.

[4] Sharma A, Gupta P, Jha R (2020). COVID-19: impact on health supply chain and lessons to be learnt. SAGE Journals.

[5] business S, generator B, address W (2021). Inventory management in 2021: techniques, formulas, software. https://www.shopify.in/retail/inventory-management.

[6] Drug Store Management (2021). BP703T_PP_V.pdf. Iptsalipur.org.

[7] Debbarma M, Rani U (2020). A review study on pharmaceutical inventory management & store keeping practices of pharmacy in rural hospitals. Indian J Public Health Res Dev.

[8] Velo GP, Minuz P (2009). Medication errors: prescribing faults and prescription errors. Br J Clin Pharmacol.

[9] Liu S, Luo P, Tang M, Hu Q, Polidoro JP, Sun S, Gong Z (2020). Providing pharmacy services during the coronavirus pandemic. Int J Clin Pharm.

[10] Iyengar K, Vaishya R, Bahl S, Vaish A (2020). Impact of the coronavirus pandemic on the supply chain in healthcare. Br. J. Health Care Manag..

[11] Kokilam MB, Kamath V (2015). Assessment of pharmaceutical store and inventory management in rural public health facilities - a study with reference to Udupi District, Karnataka. Pharm Methods.

[12] Shinde R, Kale A, Chube S, Sawant M (2017). Drug utilization study in medical intensive care unit in a rural tertiary care teaching hospital in Maharashtra. Int J Med Sci Public Health.

[13] Hussain M, Siddharth V, Arya S (2019). ABC, VED and lead time analysis in the surgical store of a public sector tertiary care hospital in Delhi. Indian J Public Health.

[14] Abelenda-Alonso G, Padullés A, Rombauts A (2020). Antibiotic prescription during the COVID-19 pandemic: a biphasic pattern. Infect Control Hosp Epidemiol.

[15] Ammassari A, Di Filippo A, Trotta MP, Traversa G, Pierantozzi A, Trotta F, Magrini N (2021). Comparison of demand for drugs used for COVID-19 treatment and other drugs during the early phase of the COVID-19 pandemic in Italy. JAMA Netw Open.

[16] Gonzalez-Zorn B (2021). Antibiotic use in the COVID-19 crisis in Spain. Clin Microbiol Infect.

[17] Grau S, Echeverria-Esnal D, Gómez-Zorrilla S (2021). Evolution of antimicrobial consumption during the first wave of COVID-19 pandemic. Antibiotics (Basel).

[18] Gharebaghi R, Heidary F, Moradi M, Parvizi M (2020). Metronidazole; a potential novel addition to the COVID-19 treatment regimen. Arch Acad Emerg Med.

[19] Yasri S, Wiwanitkit V (2020). Pain management during the COVID-19 pandemic. Pain Med.

[20] (2021). Rational use of personal protective equipment for coronavirus disease (COVID-19): interim guidance. https://apps.who.int/iris/handle/10665/331215.

[21] (2021). Personal protective equipment for COVID-19. https://www.who.int/teams/health-product-policy-and-standards/assistive-and-medical-technology/medical-devices/ppe/ppe-covid.

[22] (2021). World Health Organization. Transmission of SARS-CoV- 2: implications for infection prevention precautions. https://www.who.int/news-room/commentaries/detail/transmission-of-sars-cov-2-implications-for-infection-prevention-precautions.

